# Adult-onset macrophage activation syndrome treated by interleukin-1 inhibition

**DOI:** 10.1093/rap/rkad014

**Published:** 2023-02-01

**Authors:** Francesca Della Casa, Angelica Petraroli, Ilaria Mormile, Gianluca Lagnese, Antonio Di Salvatore, Francesca Wanda Rossi, Amato de Paulis

**Affiliations:** Department of Translational Medical Sciences, University of Naples Federico II, Naples, Italy; Department of Translational Medical Sciences, University of Naples Federico II, Naples, Italy; Center for Basic and Clinical Immunology Research, WAO Center of Excellence, University of Naples Federico II, Naples, Italy; Department of Translational Medical Sciences, University of Naples Federico II, Naples, Italy; Post-Graduate Program in Clinical Immunology and Allergy, University of Naples Federico II, Naples, Italy; Post-Graduate Program in Clinical Immunology and Allergy, University of Naples Federico II, Naples, Italy; Department of Translational Medical Sciences, University of Naples Federico II, Naples, Italy; Center for Basic and Clinical Immunology Research, WAO Center of Excellence, University of Naples Federico II, Naples, Italy; Department of Translational Medical Sciences, University of Naples Federico II, Naples, Italy; Center for Basic and Clinical Immunology Research, WAO Center of Excellence, University of Naples Federico II, Naples, Italy

Key message

- This case suggests the safety and effectiveness of anakinra as first-line biologic therapy in MAS.

Dear Editor, Secondary haemophagocytic lymphohistiocytosis (sHLH)/macrophage activation syndrome (MAS) describes a potentially life-threatening hyperinflammatory syndrome that can stem from severe infection, malignancy or autoimmune disease, most commonly systemic JIA (sJIA) and adult-onset Still’s disease (AOSD). With greater clinical recognition, an increased frequency of MAS in other systemic inflammatory disorders has been reported [1].

IL-1 inhibition assumes a central role in sHLH/MAS. Anakinra, a recombinant IL-1 receptor antagonist (IL-1Ra) has a half-life of ≈3–4 h and a good safety profile [2, 3]. It is commonly used for the management of autoinflammatory diseases, used off-label for MAS [3] and was recently approved for cytokine storm due to severe acute respiratory syndrome coronavirus 2 infection [4]. Here we report our experience with adult-onset MAS in a patient treated with high-dose anakinra.

A 31-year-old Caucasian man, healthy until the appearance of high fever, presented almost 20 days after the third dose of coronavirus disease 2019 (COVID-19) vaccination at the San Luca Hospital. An initial diagnosis of *Morbillivirus* infection, because of the high fever (>40°C), fatigue, maculopapular rash, cervical lymphadenopathy, sore throat, elevation of liver enzymes and high levels of anti-measles IgG, was excluded because of negative molecular exams. Blood tests showed anaemia [haemoglobin (Hb) 11 g/dl], thrombocytopenia (80 000/μl), hypofibrinogenemia (91 mg/dl), hypertriglyceridemia (293 mg/dl), increased CRP (171 mg/dl) and hyperferritinemia (93 000 ng/ml), with a normal ESR (8 mm/h). Radiological exams showed ascites, mild pericardial effusion and copious pleural effusion, with the need for oxygen therapy.

Based on the clinical picture and blood tests, MAS was suspected as a first manifestation of AOSD, because the patient fit the Yamaguchi criteria [5] and his HScore [6] showed a 99% probability of haemophagocytic syndrome. On hospital day (HD) 3, immunosuppressive therapy (methylprednisolone 1 g/day and ciclosporin 400 mg/day) was started. After an initial response, the patient’s symptoms and blood tests deteriorated. On HD5 he was transferred to a third-level centre, UOC of Internal Medicine and Clinical Immunology, AOU Federico II in Naples.

Blood tests at admission showed anaemia, hypofibrinogenemia, hypertriglyceridemia and a marked increase in liver enzymes, CRP, ferritin, LDH (3980 U/l), D-dimer (28 644 ng/ml), creatinine kinase (6038 U/l) and troponin (760 pg/ml), with normal electrocardiogram and echocardiography. A chest and abdomen CT scan without contrast showed pleural effusion, ascites, hepatomegaly and bilateral, symmetric focal lesions of unknown origin in the psoas muscles.

At AOU Federico II, anakinra was started at 200 mg/day i.v. on HD1 but was increased to 800 mg/day (administered as 200 mg i.v. every 6 h) on HD2 following worsening blood tests (increased ferritin and decreased haemoglobin) and deteriorating clinical condition despite high-dose glucocorticoids. Methylprednisolone 1 g/day and ciclosporin 400 mg/day were continued and, on HD3, IVIG 2 g/kg was started along with fibrinogen, enoxaparin and tranexamic acid to regulate coagulation. The patient showed a critical decrease in Hb values (5.2 g/dl) on HD3 and was transfused.

Of note, methylprednisolone 1 g/day was given for 3 days before anakinra was started and was continued for a total of 7 days.

Abdominal CT with contrast and MRI revealed symmetric focal lesions in the psoas muscle of haemorrhagic origin (Fig. 1). On HD5 the patient underwent an arterial angiography to treat any bleeding vessels. Unfortunately, no bleeding vessels were found. Therefore, octreotide therapy against occult bleeding in small vessels was initiated. In accordance with the haemostasis specialists, enoxaparin was maintained along with tranexamic acid, octreotide and fibrinogen in order to balance haemostatic function. During MAS, coagulopathy is frequent and bleeding can even occur after minor trauma [7].

**Figure 1.**
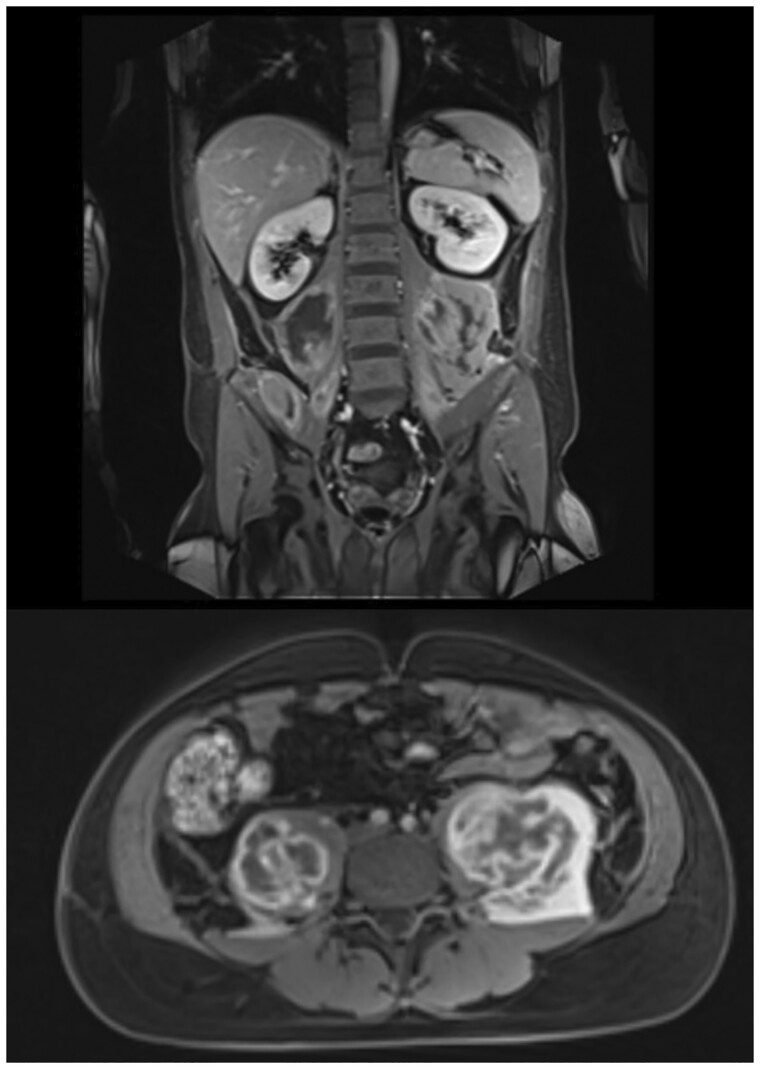
Abdominal MRI after weaning off oxygen showing haemorrhagic infarction of the psoas muscles

Another confounding factor was a positive test for EBV when the patient was admitted to San Luca Hospital [positive IgM viral capsid antigen (VCA) and IgM Epstein–Barr nuclear antigen (EBNA), IgG VCA 667 U/ml, IgG EBNA >600 U/ml], raising questions about MAS pathogenesis. Prior to this, EBV tests (IgG and IgM) were negative (performed before hospitalization, on day 7 after symptom onset).

EBV is listed among the causes of MAS, but it can also reactivate during MAS [7]. After consultation with infectiologists, the patient was diagnosed with an EBV reactivation in the context of sHLH/MAS. This diagnosis was based on clinical experience and published literature on critically ill patients with COVID-19, in which EBV and cytomegalovirus reactivation were frequent in the context of cytokine storm [8].

Finally, after 7 days of steroid boluses and 5 days of anakinra, the clinical picture improved. After 10 days of anakinra, the patient started therapy tapering until HD28, when he was discharged in good general clinical condition and with a notable improvement in blood tests (ferritin 1079 ng/ml). Therapy at discharge was s.c. anakinra 200 mg/day, prednisone 50 mg/day, ciclosporin 150 mg/day, gabapentin 300 mg/day for pain derived from muscle damage and antibiotic prophylaxis.

The patient underwent monthly follow-up for therapy tapering and monitoring of MAS reactivation for 3 months and then once every 3 months. Two months after discharge, anakinra was tapered to s.c. 100 mg/day; at 7 months, steroid therapy was tapered to prednisone 5 mg/day and ciclosporin 50 mg/day. Genetic tests for inherited immunodeficiency and autoinflammatory diseases associated with MAS development were negative.

In this case, anakinra was safe and effective as s first-line biologic therapy in MAS, even at a high dosage similar to that used in COVID-19 cytokine storm.

## Data Availability

The data underlying this article are available in the article.
